# Supplementary material for: Movi Color: fast and accurate taxonomic classification with the move structure
Source: ACM BCB. Author manuscript; Available in PMC 2026 Apr 11. (PMC13067997; doi:10.1145/3765612.3767233)
Supplement: Supplementary info [file NIHMS2157103-supplement-Supplementary_info.pdf]

A Appendix

To gather data on the potential errors our PML scoring algorithm makes, we did an empirical analysis by first running the algorithm on all positive reads on the 48-species dataset. Then, for each PML peak, we ran an exact match (via backward search) to the reference, obtaining the true set of all documents T containing the PML match. The purpose is to analyze if the documents in the run colors along each match are similar to the true set of documents containing the match. For each PML peak, we use only the scores incremented by the PML scoring algorithm along that match, and take the set of documents U with a score at least 95% the maximum score (the maximum score is equal to the length of the match). We then compute the Jaccard Index, $|T \cap U|/|T \cup U|$, and averaged them across all PML peaks above a min match length, as seen in Appendix Figure 1. For short match lengths (≤ 15), it is common for many documents to contain the substring, and the run colors found by the PML algorithm may miss some of them. However, as the matches become longer than 16, we observe Jaccard Index goes above 0.95 and approaches 1 for longer matches, indicating that the documents found by the run colors along a PML match coincide closely with the true set of documents containing that match.

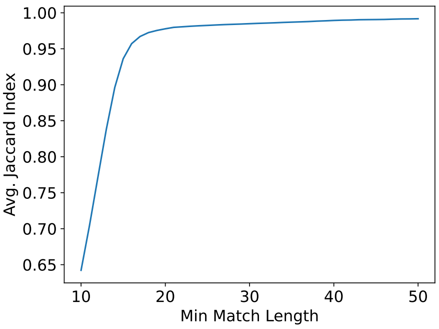

Appendix Figure 1: Jaccard Index between documents found by PML color algorithm and true documents, averaged over all PML peaks matches above a certain length.

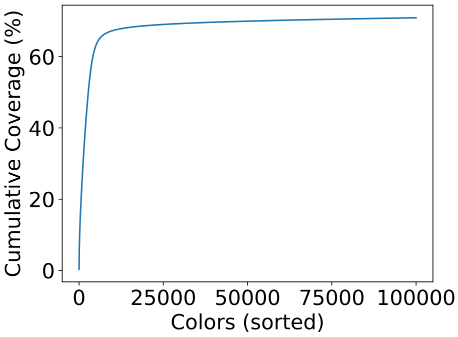

Appendix Figure 2: The cumulative coverage (% of runs) of the 10,000 most frequent colors (out of over 2.4 billion total colors) in index of *Pseudomonadota* reference genomes. The first $10,000$ **most frequent colors cover** 67.3% **of the runs.**

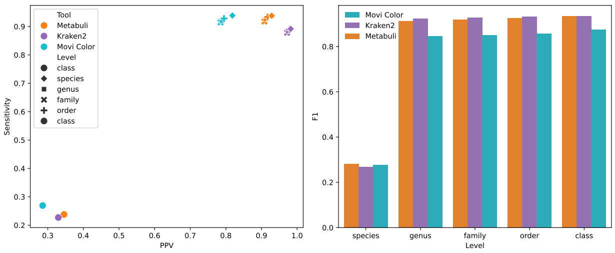

Appendix Figure 3: Classification accuracies of Movi Color compared to Metabuli and Kraken 2 on short reads. For short reads classification, Movi Color only used colors associated with PML values greater than or equal to 5.
